# Myocardial ischemia 10 years after a modified Cabrol procedure in a 42-year-old patient with Marfan syndrome

**DOI:** 10.1186/s12872-020-01740-6

**Published:** 2020-10-27

**Authors:** Ya Wei Hsiao, Jiann Woei Huang

**Affiliations:** 1 Department of Internal Medicine, Chang Gung Memorial Hospital, Keelung, Taiwan; 2 Division of Cardiovascular Surgery, Department of Surgery, Kaohsiung Medical University Hospital, Kaohsiung, Taiwan

**Keywords:** Marfan syndrome, Modified Cabrol procedure, Late complication, Myocardial infarction

## Abstract

**Background:**

Marfan syndrome, a genetic disorder of the connective tissue, may cause aortic root dilation with aortic insufficiency, aortic dissection and mitral prolapse with mitral insufficiency. We present a case of a late complication of the modified Cabrol procedure that included replacing the ascending aorta with a composite graft.

**Case presentation:**

In February 2019, a 42-year-old female patient with Marfan syndrome who presented with chest pain was sent to the Emergency Department. She had undergone the modified Cabrol procedure 10 years prior. Upon presenting, laboratory analysis revealed elevated troponin-I levels. Electrocardiogram showed new inverted T waves over lead I, aVL and V4 to V6. Contrast computed tomography (CT) revealed thrombosis in the Dacron graft. Percutaneous coronary angiography was conducted, and a large thrombus in the graft was noted. Thrombolytic therapy and percutaneous coronary intervention were performed, after which the patient had no more symptoms and was discharged without complications.

**Conclusions:**

Aortic root surgery, including the Cabrol or modified Cabrol procedure, is necessary for complicated cases of aortic dilations, such as in patients with Marfan syndrome, even though the Cabrol or modified Cabrol procedure has a high complication rate. Regarding this case, we were surprised by the timing of the myocardial ischemia and the position of the thrombus, which differed from other cases. To better manage such a patient’s condition and to detect the formation of thrombus early, completeness of the graft and possible stenosis of the anastomosis site to avoid preventable myocardial ischemia, we suggest that patients should have regular image follow-up, even years after the Cabrol or modified Cabrol procedure.

## Background

Marfan syndrome is a disorder caused by an autosomal dominant genetic variation of the connective tissue. Since connective tissue is found over the whole body, Marfan syndrome is associated with a broad range of related disorders and can present with various severities. Ocular, cardiovascular, and musculoskeletal abnormalities, even involving the central nervous system, can occur. Among all complications, aortic root dilation with aortic insufficiency, aortic dissection and mitral prolapse with mitral insufficiency are common cardiovascular conditions in Marfan syndrome [1]. The modified Cabrol procedure is one of the techniques.

for composite replacement of the aortic valve and ascending aorta. One of the Cabrol modifications that has gained popularity is reimplanting either the right or left coronary ostium with a separate interposition graft. The Cabrol graft procedure and its modifications can be safely utilized for complex aortic root replacement procedures. According to the study of Ziganshin et al., the long-term patency of the Dacron interposition grafts is 100%, as radiographically confirmed, and according to their statistics, no late deaths were related to the Cabrol interposition graft. However, there have been reports mentioning occlusion of the Cabrol graft and stenosis of the graft-coronary anastomosis. Here, we present a case of a 42-year-old female who experienced acute myocardial ischemia 10 years after the modified Cabrol procedure. The patient was successfully treated with a percutaneous coronary intervention and discharged without complications [2, 3].

## Case presentation

**Fig. 1:**
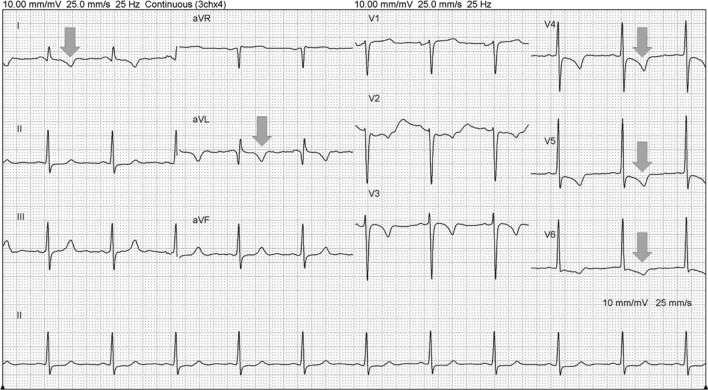
Electrocardiogram on arrival revealed inverted T waves over lead I, aVL and V4–V6

A 42-year-old woman with Marfan syndrome experienced acute chest pain while asleep and was sent to the Emergency Department of National Taiwan University Hospital-YunLin Branch at midnight. Ten years prior, the patient underwent the modified Cabrol procedure due to an aortic root aneurysm at Kaohsiung Medical University Hospital. The procedure interposed a 25-mm St. Jude’s mechanical aortic valve for progressively severe aortic regurgitation, a 28-mm graft for ascending aorta dilation and a button-in for the orifice of the right coronary artery to the 28-mm composite graft. An 8-mm-diameter Dacron graft was anastomosed end-to-side to the composite graft and end-to-end to the orifice of the left main coronary artery. The patient had no complications for 10 years after the operation. Anticoagulation therapy with warfarin sodium was started on the first postoperative day, and the target international normalized ratio was 1.8–2.5. In the latest follow-up, 1 month prior to this chest pain episode, echocardiography showed normal wall motion of the patient’s heart. Due to the previous medical history, she was transferred to Kaohsiung Medical University Hospital. Electrocardiogram on arrival showed inverted T waves over lead I, aVL and V4–V6 (Fig. 1). Initial cardiac biomarkers showed elevated troponin I of 14.89 ng/mL, CPK of 1183 IU/L and CK-MB of 138 ng/mL. She was diagnosed with acute non-ST-elevation myocardial infarction.

**Fig. 2:**
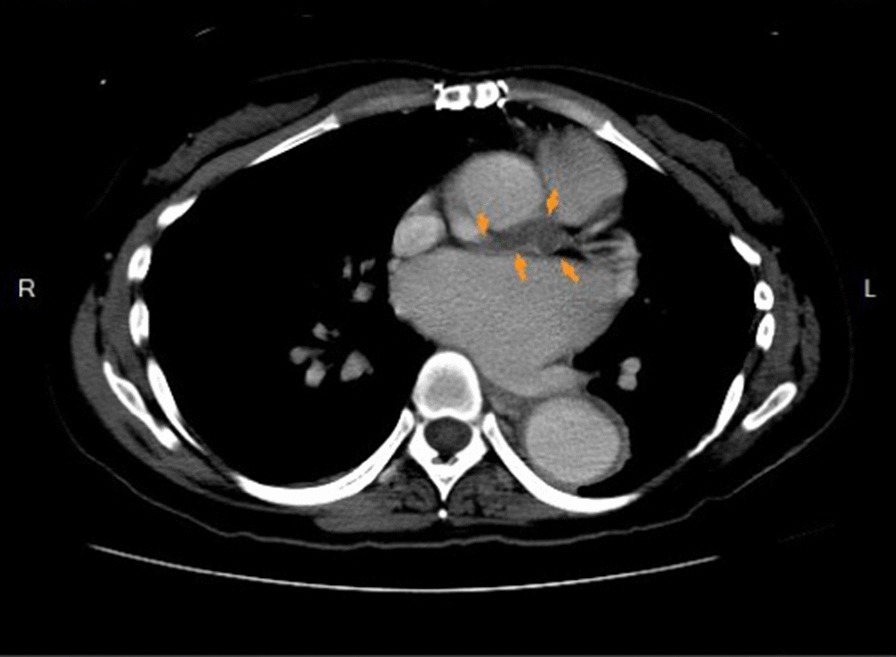
Contrast CT showed a thrombosis in the Dacron graft (Ar-row)

Contrast computed tomography revealed a thrombosis in the Dacron graft (Fig. 2). Due to persistent chest discomfort and the above findings, coronary artery occlusion was highly suspected, even though she had no risk factors, such as hypertension, hyperlipidemia, diabetes mellitus or smoking.

**Fig. 3:**
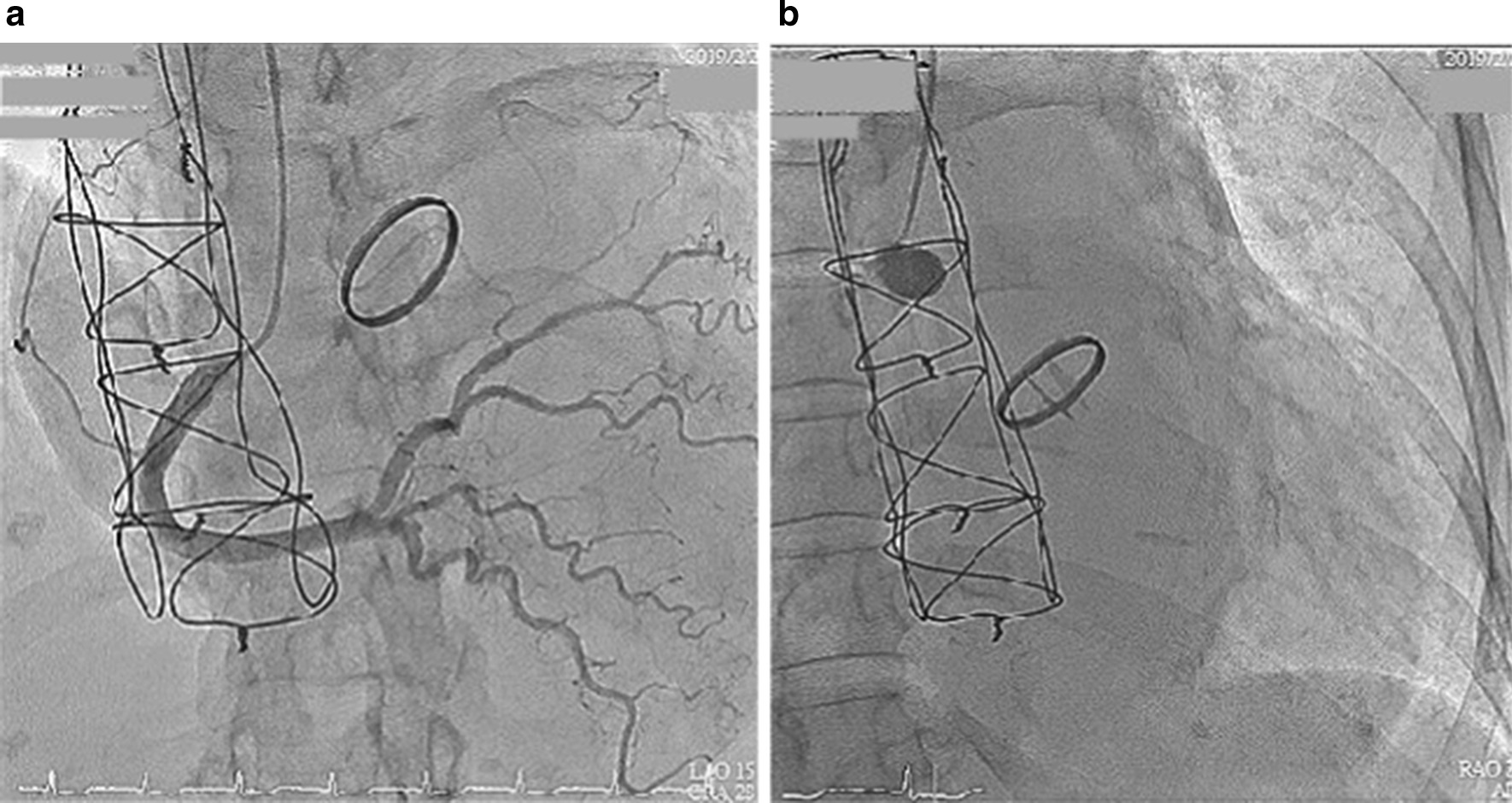
Coronary angiography showing **a** a patent right coronary artery collateral to the LAD and LCX and **b** 100% stenosis in the left main artery

**Fig. 4:**
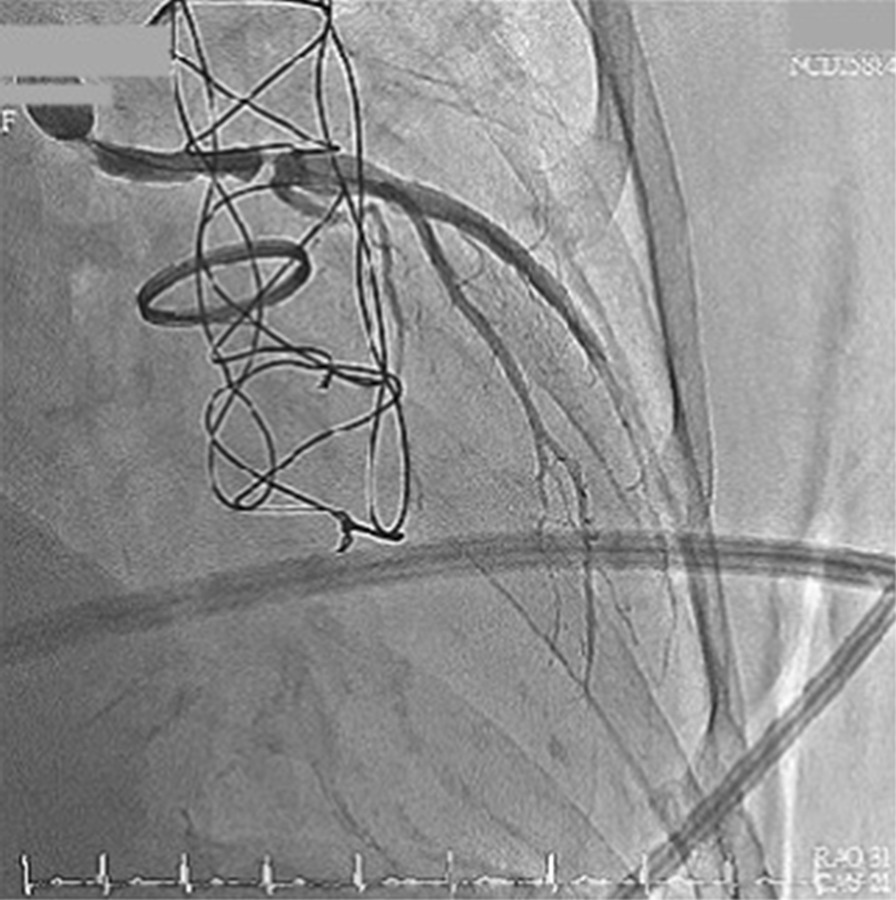
Status after thrombus aspiration and ballooning. The final angiography showed a residual thrombus in the graft between the LM orifice and distal LM

**Fig. 5:**
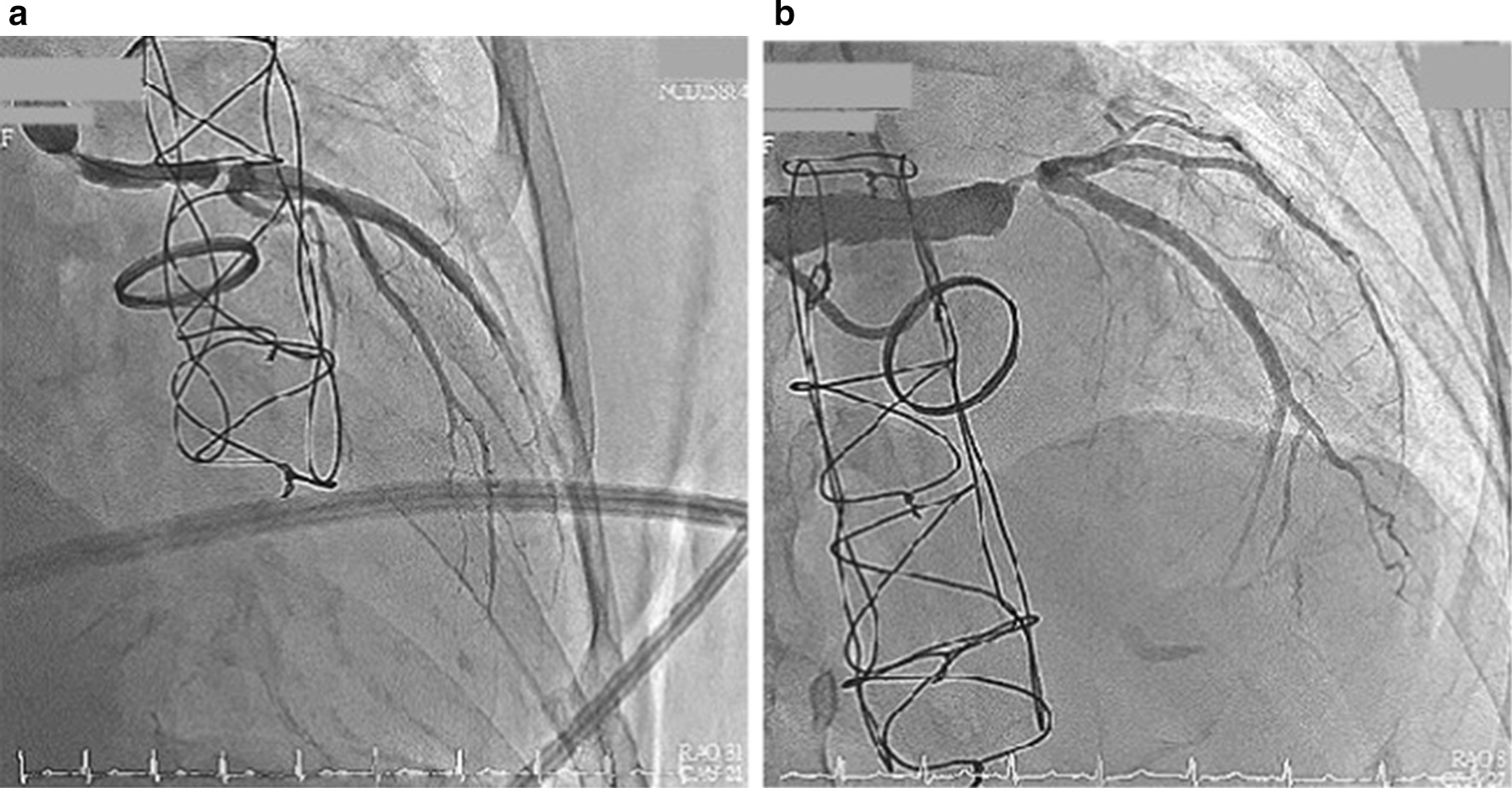
The patency of the Dacron graft **a** before and **b** after the thrombolytic therapy

**Fig. 6:**
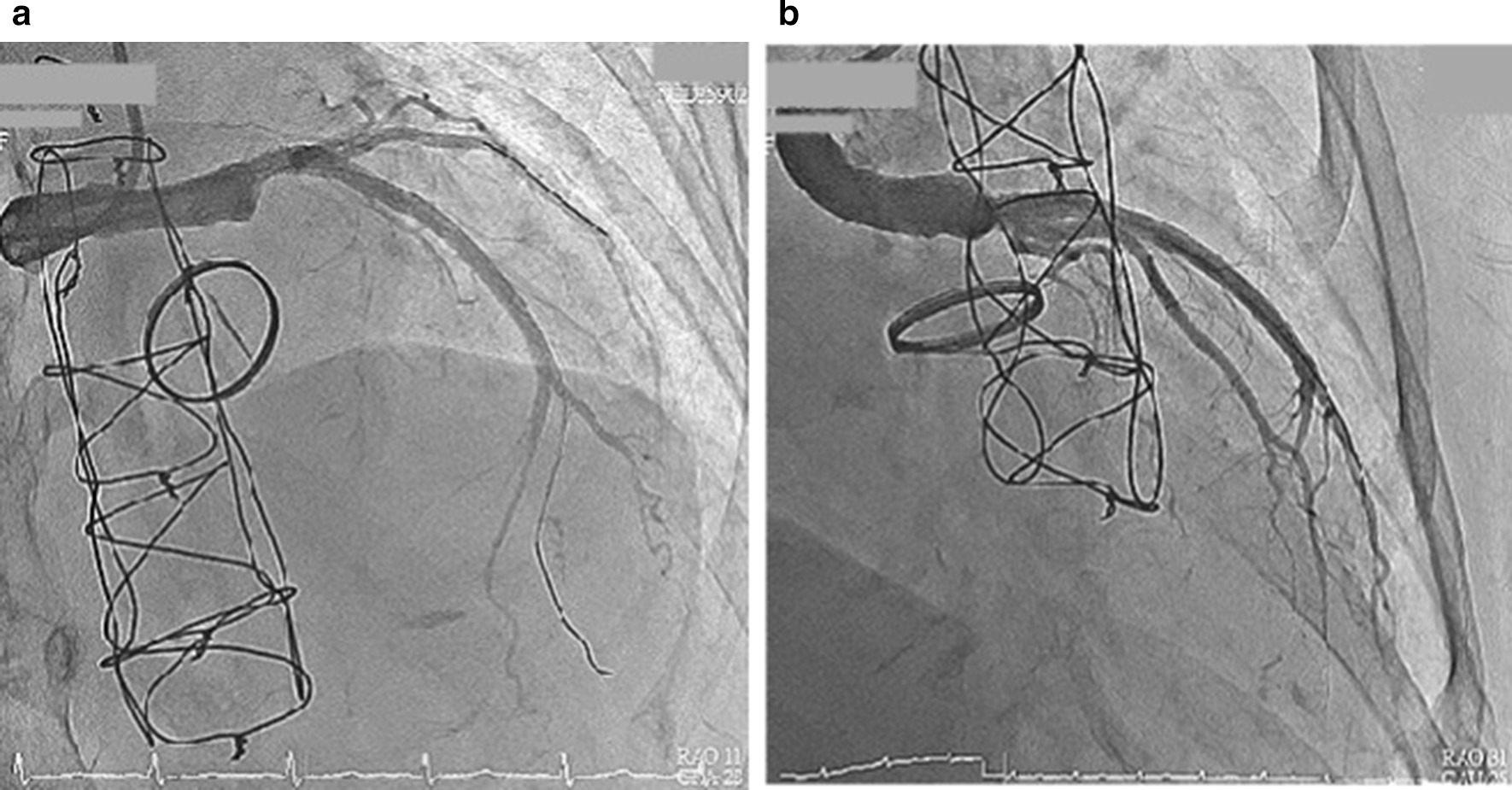
**a** The coronary angiography after stent implantation. **b** Final angiography revealed an optimal angiographic result with TIMI grade 3 flow

We instantly arranged a percutaneous coronary angiography, and many thrombi were found in the graft. Left main artery (LM) 100% stenosis and slow flow were both also noted in the left descending artery (LAD) and left circumflex artery left circumflex artery (LCX) (Fig. 3). The right coronary artery was patent and collateral to the LAD and LCX. We used an Export aspiration catheter to the LAD and LCX for intracoronary suction. After thrombus aspiration, blood flow to the LAD and LCX improved. We further utilized an Accuforce balloon of 2.0 × 15 mm to dilate the LM distal to the LAD with a maximum of 16 atm. Despite improvement of the blood flow from the graft to the LM, LAD and LCX, residual thrombi remained in the graft between the orifice of the LM and distal LM (Fig. 4). The anastomosis site of the Dacron graft and natural left main coronary artery was uncertain because of the residual thrombus after aspiration during the first percutaneous coronary intervention (PCI); therefore, the placement of the stent was intervened. In addition, as the diameter of the Dacron graft (8 mm) and the natural left main coronary artery (4–5 mm) were different, the size of the stent was unclear. For the above reasons, thrombolytic therapy and a second PCI were chosen despite a lack of previous experience. After PCI, thrombolytic therapy was initiated with urokinase for 3 days and heparin continuous infusion for 2 days. Warfarin sodium was maintained, and the target international normalized ratio was 2.0–2.5. The second angioplasty was arranged for 6 days later, and there were no complications during this period. Thrombolytic agents successfully dissolved much of the thrombi in the graft; thus, the second percutaneous coronary intervention for LM was performed with no issues (Fig. 5). Predilation was performed using a 5.0 × 15-mm NC Euphoral, and a 5.0 × 15-mm Onyx (Drug-eluting stent) was deployed at the LM. The entire lesion was dilated using a 5.0 × 15-mm NC Euphoral. An optimal angiographic result was obtained with thrombolysis in myocardial infarction (TIMI) grade 3 flow (Fig. 6). The patient was then stabilized in the intensive care unit.

Considering that the patient was 42 years old and that she had no hypertension or prior bleeding history, bleeding risk was regarded as low, however, the patient’s prior stent thrombosis indicated a high thrombotic risk. Based on the above, we prescribed dual therapy with oral form anticoagulants (warfarin sodium) and antiplatelet agents (ticagrelor) after the stentings. She was discharged five days after the second PCI without complications.

## Discussion and conclusions

Implanting composite valve grafts as treatment for a diseased aortic valve and root dilation with reattachment of the coronary arteries by a Dacron graft has been proven to offer good long-term results. Various surgical treatment modalities have been developed to perform the surgery, such as the Bentall operation, button technique, Cabrol procedure and modified Cabrol procedure. However, when dealing with such fragile, very acutely dissected or remote coronary ostial walls, many surgeons prefer the Cabrol or modified Cabrol procedure because the bleeding sites can be easily visualized and obtaining hemostasis can be easier. Additionally, there is a low incidence of false aneurysm formation at the coronary ostia [4]. Nonetheless, the Cabrol and Cabrol-related procedures are technically more complicated as a result of the necessity to have the correct diameter, length and orientation of the graft to avoid late complications, including coronary limb kinking. Occlusion of Dacron grafts results in acute myocardial infarction (AMI) or even sudden death [5].

Intraoperative and postoperative myocardial infarctions occur at an incidence of approximately 5% when aortic root reconstruction is carried out by an experienced surgeon [6], but complications of coronary reimplantation are considered more common with the Cabrol procedure. This may be related to patient characteristics because the Cabrol procedure is usually applied in the most complicated cases. According to the series of Di Marco et al., the myocardial infarction occurrence rate is 2.7% [28/1045], with a higher incidence in patients who undergo the Cabrol procedure than those treated with another approach (12.5% [3/24] vs 2.4% [25/1021]) [7]. To date, several studies have shown that most AMI cases occur early postoperatively, and kinking of the limbs of the coronary Dacron graft is viewed as the major cause [3, 4, 9, 11–13]. Overall, there were very few cases of AMI as long-term complications. Kitamura et al. examined 18 patients by either angiography or multidetector computed tomography (MDCT) for assessment of coronary grafts in long-term follow-up. Among these patients, one had an occlusion of the right coronary ostium, as shown by angiography, 186 months after the operation; another patient had stenosis in the right coronary ostium, as revealed by MDCT, 81 months after the operation [8]. The etiologies of thrombus formation or stenosis can be divided into 3 possible mechanisms, as follows. First, the most common cause of myocardial infarction is kinking. Due to interposition tube graft looping between the coronary ostia and the posterolateral part of the composite valve conduit, kinking of the graft is a susceptible reason that results in insufficient blood supply to the heart and thus causes ischemia. Second, there is a hypothesis indicating that retrograde cannula of cardioplegia during the operation might cause early intimal hyperplasia and iatrogenic small intimal tears in the coronary ostium. Third, although some might consider it unlikely to be due long-term etiology of AMI with the Cabrol or modified Cabrol procedure, the difference between the size of the 8-mm Dacron grafts and natural coronary arteries might cause a swirling flow at the orifice of the coronary artery. The ostial intimal hyperplasia resulting from turbulent flow might be the reason for late anastomotic stenosis of a coronary graft [9].

Long-term Dacron interposition graft patency has been questioned. Ziganshin et al. used computed tomography scans for radiologic follow-up. Among 31 patients, 27 survived, and 4 expired from reasons unrelated to the Cabrol procedure. Among the Dacron grafts that were evaluated radiologically, 100% showed wide patency with no mural thrombus formation. No signs of stenosis or occlusion were detected [2].

Regarding the thrombosis or stenosis site of the Dacron graft, several studies have revealed that anastomosis with the right coronary artery ostium is more vulnerable to kinking, postoperative stenosis, and occlusion. Knight et al. conducted computational research on flow dynamics and found a twirled blood stream with low flow toward the right coronary artery in a Cabrol graft and a higher incidence of occlusions of the right compared with the left Cabrol graft in long-term follow-up [10].

This is the first report of Marfan syndrome in which thrombus developed in a nonkinked left modified Cabrol graft 10 years after the operation. Successful stenting of the graft was performed, and the patient was discharged uneventfully after PCI. This highlights the importance of long-term awareness of complications when encountering patients who have undergone coronary reimplantations with the Cabrol or modified Cabrol procedure. We suggest that noninvasive methods, such as computed tomography, magnetic resonance imaging or transesophageal echocardiography, be routinely utilized for postoperative follow-up in patients who receive composite graft replacements for early detection of thrombosis or stenosis.

## Data Availability

Data generated or analyzed during this study are included in this published article.

## Abbreviations

AMI: Acute myocardial infarction
CT: Computed tomography
LAD: Left descending artery
LCX: Left circumflex artery
LM: Left main artery
MDCT: Multidetector computed tomography
PCI: Percutaneous coronary intervention
TIMI: Thrombolysis in myocardial infarction

## References

[1] Pyeritz RE, Mckusick VA (1979). The Marfan syndrome: diagnosis and management. N Engl J Med.

[2] Ziganshin BA, Williams FE, Tranquilli M, Elefteriades JA (2014). Midterm experience with modified Cabrol procedure: Safe and durable for complex aortic root replacement. J Thorac Cardiovasc Surg.

[3] Jang WI, Jeong JO, Ahn KT, Park HS, Park JH, Kim SS, Lee JH, Choi SW, Seong IW (2010). A successful primary percutaneous coronary intervention twelve days after a Cabrol composite graft operation in Marfan syndrome. Korean Circ J.

[4] Çetin G, Özkara A, Tireli E, Köner Ö, Süzer K (2005). Myocardial ischemia after Cabrol operation. Asian Cardiovasc Thorac Ann.

[5] Jault F, Nataf P, Rama A, Fontanel M, Vaissier E, Pavie A (1994). Chronic disease of the ascending aorta. Surgical treatment and long-term results. J Thorac Cardiovasc Surg.

[6] Etz CD, Bischoff MS, Bodian C, Roder F, Brenner R, Griepp RB, Luozzo GD (2010). The Bentall procedure: is it the gold standard? A series of 597 consecutive cases. J Thorac Cardiovasc Surg.

[7] Marco LD, Pacini D, Pantaleo A, Leone A, Barberio G, Marinelli G, Bartolomeo RD (2016). Composite valve graft implantation for the treatment of aortic valve and root disease: results in 1045 patients. J Thorac Cardiovasc Surg.

[8] Kitamura T, Kigawa I, Fukuda S, Miyairi T, Takamoto S (2011). Long term results with the Cabrol aortic root replacement. Int Heart J.

[9] Lin HC, Kuo CH, Li JY, Hou Charles JY, Chou Y-S, Tsai CH (2003). A rare late complication following composite graft replacement of aortic root in a patient with Marfan syndrome: a case report and literature review. J Intern Med Taiwan.

[10] Knight J, Baumüller S, Kurtcuoglu V, Turina M, Turina J, Schurr U, Poulikakos D, Marshall W, Alkadhi H (2010). Long-term follow-up, computed tomography, and computational fluid dynamics of the Cabrol procedure. J Thorac Cardiovasc Surg.

[11] Sekine S, Abe T, Seki K, Shibata Y, Yamagishi I, Kamada M (1995). Dacron coronary graft obstruction after composite graft replacement of aortic root. Ann Thorac Surg.

[12] Witzenbichler B, Schwimmbeck P, Schultheiss H-P (2005). Myocardial infarction caused by occlusion of Cabrol conduit graft. Circulation.

[13] Hoskins MH, Kacharava AG, Green TF, Mavromatis K (2010). Percutaneous intervention of Cabrol graft-left main anastomosis during acute myocardial infarction. Int J Cardiol.

